# Enzymes from Extreme Environments and Their Industrial Applications

**DOI:** 10.3389/fbioe.2015.00161

**Published:** 2015-10-13

**Authors:** Jennifer A. Littlechild

**Affiliations:** ^1^Exeter Biocatalysis Centre, Biosciences, College of Life and Environmental Sciences, University of Exeter, Exeter, UK

**Keywords:** γ-lactamase, l-aminoacylase, transaminase, carbonic anhydrase, epoxide hydrolase, esterase, dehalogenase

## Abstract

This article will discuss the importance of specific extremophilic enzymes for applications in industrial biotechnology. It will specifically address those enzymes that have applications in the area of biocatalysis. Such enzymes now play an important role in catalyzing a variety of chemical conversions that were previously carried out by traditional chemistry. The biocatalytic process is carried out under mild conditions and with greater specificity. The enzyme process does not result in the toxic waste that is usually produced in a chemical process that would require careful disposal. In this sense, the biocatalytic process is referred to as carrying out “green chemistry” which is considered to be environmentally friendly. Some of the extremophilic enzymes to be discussed have already been developed for industrial processes such as an l-aminoacylase and a γ-lactamase. The industrial applications of other extremophilic enzymes, including transaminases, carbonic anhydrases, dehalogenases, specific esterases, and epoxide hydrolases, are currently being assessed. Specific examples of these industrially important enzymes that have been studied in the authors group will be presented in this review.

## Introduction

A problem with using enzymes for industrial biotransformation reactions is often their inherent stability to the conditions employed. During industrial processes, the enzymes are often exposed to a different environment to their natural conditions within the cell such as non-natural substrates, high substrate concentrations, non-aqueous conditions, and extremes of pH. When using non-natural substrates, enzymes from different classes can be found to accept the same substrate such as the case with racemic γ-lactam described below. Enzymes can also carry out “side reactions” when used at a different pH than that found inside the cell.

The availability of new genome sequences makes the search for new industrial enzymes a relatively easy process. Also the isolation of metagenomes from extremophilic sources provides DNA from potentially uncultivatable organisms. However, the identification of specific enzymes from this resource is only as good as the current bioinformatic analyses and many novel or unknown activities can be missed. It is therefore important to also screen genomic libraries against substrates of commercial interest for specific biocatalytic activities especially if turnover of a non-natural substrate is required.

The diversity which “nature” provides for extremophilic environments of temperature, pH, acidity, alkalinity, pressure, and salinity can be exploited to discover new novel and potentially robust enzymes that are better suited for use in industrial applications. The increased number of extremophilic genomes and metagenomes that can now be sequenced by next-generation sequencing technologies provides an ever expanding resource for identification of new enzymes.

The mechanism of protein stabilization under extreme conditions varies depending on the microbial species and level of adaption required for survival in the host organism. For the acidophiles and alkalophiles, it is only the proteins exported from the cell that have to be stable under the extreme pHs of the growth environment, since the proteins inside the cell do not have to withstand these extreme conditions as the intracellular pH is maintained around pH 5.0–6.0. Some general features of enzyme stability have been observed from the analysis of the three-dimensional structures of enzymes isolated from extreme environments of high temperatures have been reviewed by Littlechild et al. (2013).

Many archaeal and bacterial enzymes isolated from extremophiles have general applications in molecular biology such as the hyperthermophilic *Pyrococcus* polymerase enzyme which has improved fidelity in PCR reactions when compared to thermophilic bacterial polymerase enzymes. Other thermophilic enzymes are of great importance to the breakdown of biomass and other materials such as waste plastics in order to contribute to a circular economy where nothing is wasted.

This review will concentrate on several specific examples of interest to the author where extremophilic enzymes are currently playing an important role as biocatalysts for the pharmaceutical and fine chemical industries.

## Specific Examples

### The *Sulfolobus solfataricus* γ-Lactamase Enzyme

An enzyme from the thermophilic archaeon *Sulfolobus solfataricus* MT4 can use the bicyclic synthon (rac)-γ-lactam (2-azabicyclo[2.2.1]hept-5-en-3-one) as a substrate to obtain a single enantiomer of the γ-bicyclic lactam product which is an important building block for the anti-HIV compound, Abacavir (Taylor et al., 1993). This (+)-γ-lactamase was identified in the *Sulfolobus* strain by screening colonies from an expression library for their ability to produce the amino acid product when supplied with the racemic γ-lactam. Screening was carried out using genomic libraries using a filter paper overlay. The colonies on the plate that were active showed a brown coloration of the filter paper when the amino acid was produced which had been soaked in ninhydrin stain. Another non-thermophilic bacterial (+)-γ-lactamase that can also carry out this reaction has been identified within the bacterial *Delftia* species (PDB code 2WKN, Gonsalvez et al., 2001). This enzyme is of a different class, structure, and mechanism from the archaeal enzyme but both can use the non-natural γ-lactam as a substrate. It is related to the bi-zinc containing metalloprotease formamidase by analysis using the SCOP2 database (Andreeva et al., 2014). A third (−)-γ-lactamase of opposite stereoselectivity is related to a α/β hydrolase fold esterase enzyme which can also carry out the side reaction of bromination at pH 4.0 and was referred to as a non-cofactor containing bromoperoxidase (Line et al., 2004).

The *Sulfolobus* (+)-γ-lactamase has been cloned and overexpressed in *Escherichia coli* and purified to homogeneity (Toogood et al., 2004). The molecular mass of the monomer was estimated to be 55 kDa by SDS-PAGE, which is consistent with the calculated molecular mass of 55.7 kDa. The native molecular weight was 110 kDa as determined by gel filtration chromatography, indicating that the enzyme exists as a dimer in solution. The purified enzyme has been crystallized with a view to determining its three-dimensional structure.

This thermostable archaeal (+)-γ-lactamase has a high sequence homology to the signature amidase family of enzymes. It shows similar inhibition to the amidase enzymes with benzonitrile, phenylmethylsulfonyl fluoride, and heavy metals such as mercury, and it is activated by thiol reagents. Alignment of the amino acid sequences of the (+)-γ-lactamase from *S. solfataricus* with four amidases from *Pseudomonas chlororaphis* B23, *Rhodococcus* sp. N-771, *Rhodococcus erythropolis* N-774, and *Rhodococcus rhodochrous* J shows it has a 41–44% sequence identity to these enzymes. The amidases belong to the signature amidase family as they all contain the consensus sequence GGSS(S/G)GS. The amino acid sequence of the γ-lactamase contains the highly conserved putative catalytic residues aspartic acid and serine but not the highly conserved cysteine residue (Kobayashi et al., 1997).

The purified (+)-γ-lactamase enzyme has been immobilized as a cross-linked, polymerized enzyme preparation and packed into microreactors (Hickey et al., 2009). The thermophilic (+)-γ-lactamase retained 100% of its initial activity at the assay temperature, 80°C, for 6 h and retained 52% activity after 10 h, indicating the advantage of the immobilization. The free enzyme began to display a reduction in activity after 1 h at 80°C. The higher stability of the immobilized enzyme provided the advantage that it could be used to screen many compounds in a microreactor system without denaturation.

### l-Aminoacylase and Pyroglutamyl Carboxyl Peptidase from the Thermophilic Archaeon *Thermococcus litoralis*

**Figure 1:**
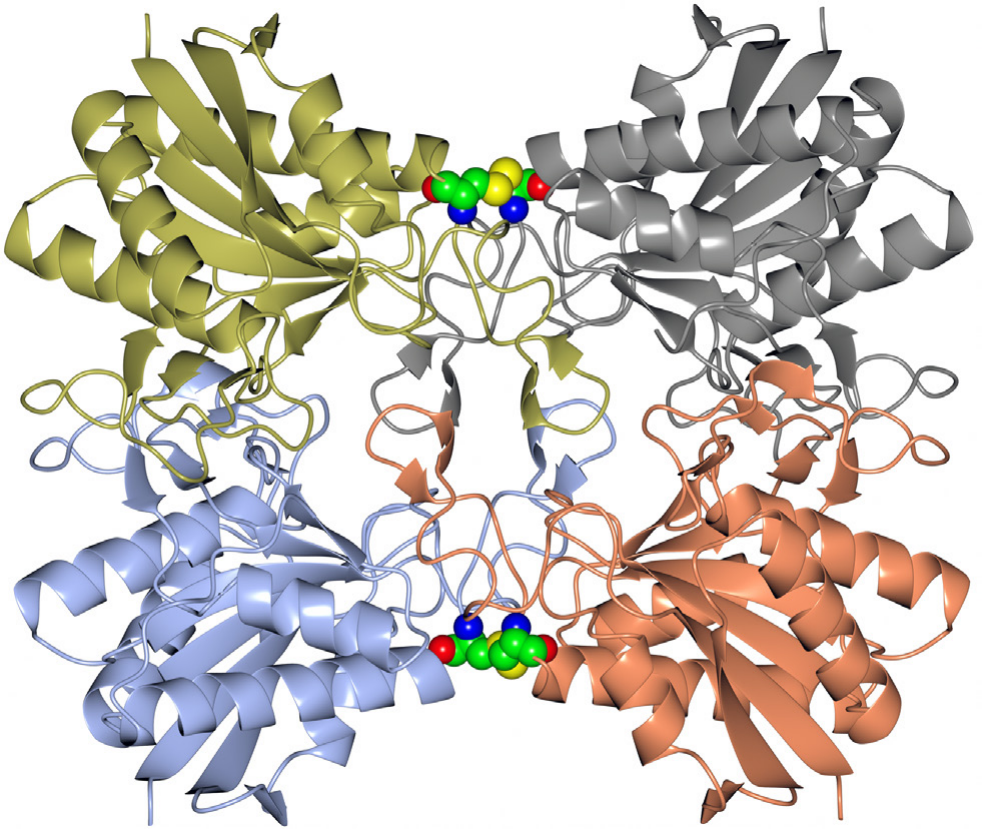
**A diagram showing the tetrameric structure of the *Thermococcus* pyroglutamyl carboxyl peptidase**. The secondary structural elements are shown in a different color for each subunit. The hydrophobic core structure is shown in the center of the molecule and the disulfide bonds at the subunit interfaces in sphere mode (Singleton et al., 1999, PDB code 1A2Z).

Many pharmaceutically active compounds contain nitrogen and can be derived from amino acids (Drauz, 1997). These compounds need to be optically pure, which can be achieved by the use of specific l or d aminoacylase enzymes. There is a large growth in the use of unnatural amino acids, for example, l-*tert*-leucine is a precursor to many pharmaceutically active compounds such as the antitumor compounds (Bommarius et al., 1998). A thermophilic archaeal l-aminoacylase has been cloned and overexpressed from the archaeon *Thermococcus litoralis* (Toogood et al., 2002a). The enzyme was identified from a *Thermococcus* DNA expression library which gave a positive hit for esterase activity. This esterase gene was later found to code for a pyroglutamyl carboxyl peptidase, which is a novel cysteine protease that cleaves the pyroglutamyl group from the N-terminus of biologically important peptides. The enzyme has been characterized both biochemically and structurally and demonstrated to be a new class of cysteine protease (Singleton et al., 1999). The commercial use of this enzyme is to cleave the pyroglutamyl group from “blocked” peptides allowing them to be N-terminally sequenced. The *Thermococcus* protease forms a tetrameric structure held together by disulfide bonds between the dimer subunit interface and is stabilized by an unusual hydrophobic core at the center of the tetrameric structure. This unusual feature is formed from four two-stranded antiparallel β-sheets, one from each subunit. The sheets are built from the hydrophobic amino acid residues Phe–Phe–Leu–Leu (Figure 1). This hydrophobic insertion is unique to the *T. litoralis* enzyme, with no equivalent structure seen in other bacterial or archaeal pyroglutamyl carboxyl peptidase enzymes (Singleton et al., 1999).

The *Thermococcus* l-aminoacylase enzyme which was identified next to the novel cysteine protease and has an 82% sequence identity to an l-aminoacylase from *Pyrococcus furiosus* (Tanimoto et al., 2008) and 45% sequence identity to a carboxypeptidase from another thermophilic archaeon *S. solfataricus*. The *Thermococcus* enzyme is not inhibited by conventional aminoacylase inhibitors such as mono-*tert*-butyl malonate so appears to be novel. It is thermostable, with a half-life of 25 h at 70°C and has an optimal activity at 85°C in Tris-HCl, pH 8.0. This *T. litoralis* l-aminoacylase has a broad substrate specificity preferring the amino acids: Phe ≫ Met > Cys > Ala ≃ Val > Tyr > Propargylglycine > Trp > Pro > Arg. A column bioreactor containing the recombinant *Thermococcus* enzyme has been constructed by immobilization onto Sepharose beads. This bioreactor showed no loss of activity towards the substrate *N*-acetyl-dl-Trp after 5 days and 32% of the activity remained after 40 days at 60°C (Toogood et al., 2002b). The enzyme has more recently been immobilized by covalent attachment to an epoxy resin formed in channels of a microreactor allowing a “flow-through” procedure to be used (Ngamsom et al., 2010). This can be used for rapid substrate screening of the l-aminoacylase and eliminates potential substrate and product inhibition. It has been shown in pilot-scale biotransformation reactions using the substrate *N*-acetyl-dl-propargylglycine that this enzyme does show substrate inhibition (Toogood et al., 2002a).

The *Thermococcus* l-aminoacylase enzyme is now being used for multiton commercial production of l-amino acids and their analogs by Chirotech/Dow Pharma and more recently by Chirotech/Dr Reddys (Holt, 2004). A racemase enzyme has been developed in order to convert the isomer not used by the enzyme to the form that is used which can enable a more efficient process resulting in a 100% conversion of the racemic substrate (Baxter et al., 2012).

There are differences in substrate specificity between the *Thermococcus* l-aminoacylase and another thermophilic archaeal enzyme from *Pyrococcus* species. The substrate *N*-acetyl-l-phenylalanine is the most favorable substrate for the *Thermococcus* enzyme; however, this substrate is not used by the *Pyrococcus* l-aminoacylase (Tanimoto et al., 2008).

### Carboxyl Esterase from a Thermophilic Bacterium *Thermogutta terrifontis*

**Figure 2:**
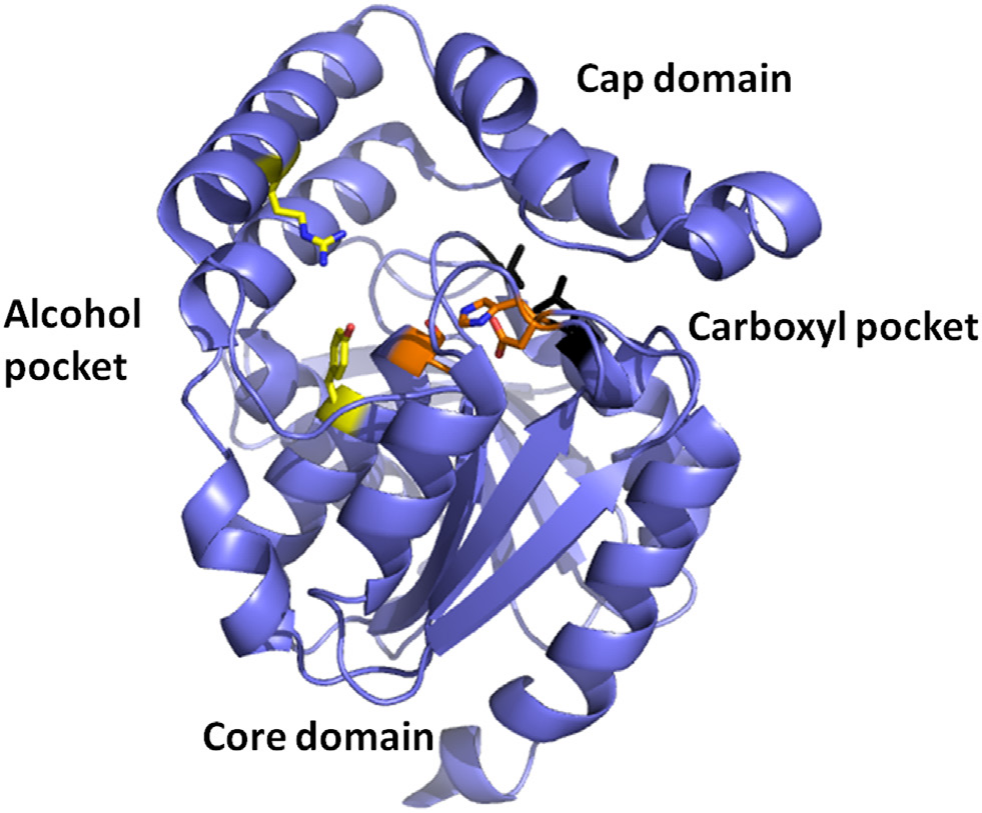
**A cartoon representation of the *Thermogutta* carboxyl esterase structure showing the active site residues in stick mode between the cap and the core domain**. The binding pockets for the carboxyl group and the alcohol groups of the para nitrophenol ester substrate are also highlighted (Sayer et al., 2015, PDB code 4UHC).

Esterases are a class of commonly used enzymes in industrial applications. This is partially due to their inherent stability in organic solvents and the ability to freely reverse the enzyme reaction from hydrolysis to synthesis by the elimination of the water that is used during the hydrolysis mechanism. The carboxyl esterases catalyze the hydrolysis of the ester bond of relatively small water-soluble substrates. A new carboxyl esterase (TtEst) has been identified in a recently identified thermophilic bacterium *Thermogutta terrifontis* from the phylum *Planctomycetes*. This enzyme has been cloned and overexpressed in *E. coli* (Sayer et al., 2015). The enzyme has been characterized biochemically and shown to have activity towards small *p*-nitrophenyl (*p*NP) carboxylic esters with optimal activity for *p*NP-propionate. The TtEst enzyme is very thermostable and retained 95% of its activity after incubation for 1 h at 80°C. The enzyme has been crystallized and its structure determined without ligands bound in the active site and in complex with a substrate analog d-malate and the product acetate. The bound ligands in the structure have allowed the identification of the carboxyl and alcohol binding pockets in the enzyme active site (Figure 2). It has also allowed a detailed comparison with structurally related enzymes that has given insight into how differences in the catalytic activity can be rationalized based on the properties of the amino acid residues in different active site pockets. An overall comparison of the alcohol binding pocket in TtEst with the equivalent pocket in the 3-oxoadipate-enol lactonase from *Burkholderia xenovorans* (PcaD) with 29% sequence identity (PDB code 2XUA) (Bains et al., 2011) and *Aureobacterium* species (−) γ-lactamase (Agl) with 30% sequence identity (PDB code 1HKH) (Line et al., 2004) has been carried out. The catalytic triad residues and the position of the oxyanion hole are conserved between these enzymes. The PcaD and Agl show that the TtEst pocket has a much more polar and charged environment in the active site, which allows the binding of organic acids such as d-malate where the distant carboxyl is coordinated by Arg139 and Tyr105. The PcaD and Agl enzymes have more hydrophobic substrate binding pockets, with the residues Arg139 and Tyr105 in TtEst replaced by Trp135 and Ile129 in PcaD and Trp204 and Leu125 in Agl. In both PcaD and Agl, the active site is better suited for the binding of structures such as lactone and γ-lactam rings. Similarly, the *Pseudomonas fluorescens* esterase with 30% sequence identity (PDB code 3HEA) (Yin et al., 2010) has an alcohol pocket where the structure is lined with several hydrophobic phenylalanine side chains that should have affinity for the lactone ring. This would explain its lactonase activity towards caprolactone (Cheeseman et al., 2004) in addition to the esterase activity.

Mutant enzymes have been constructed to extend the substrate range of *T. terrifontis* esterase to accept the larger butyrate and valerate *p*NP esters. These mutant enzymes have also shown a significant increase in activity towards acetate and propionate *p*NP esters. A crystal structure of the Leu37Ala mutant has been determined with the butyrate product bound in the carboxyl pocket of the active site. The mutant structure shows an expansion of the pocket that binds the substrate carboxyl group, which is consistent with the observed increase in activity towards *p*NP-butyrate.

### A α-Carbonic Anhydrase from the Thermophilic Bacterium *Thermovibrio ammonificans*

**Figure 3:**
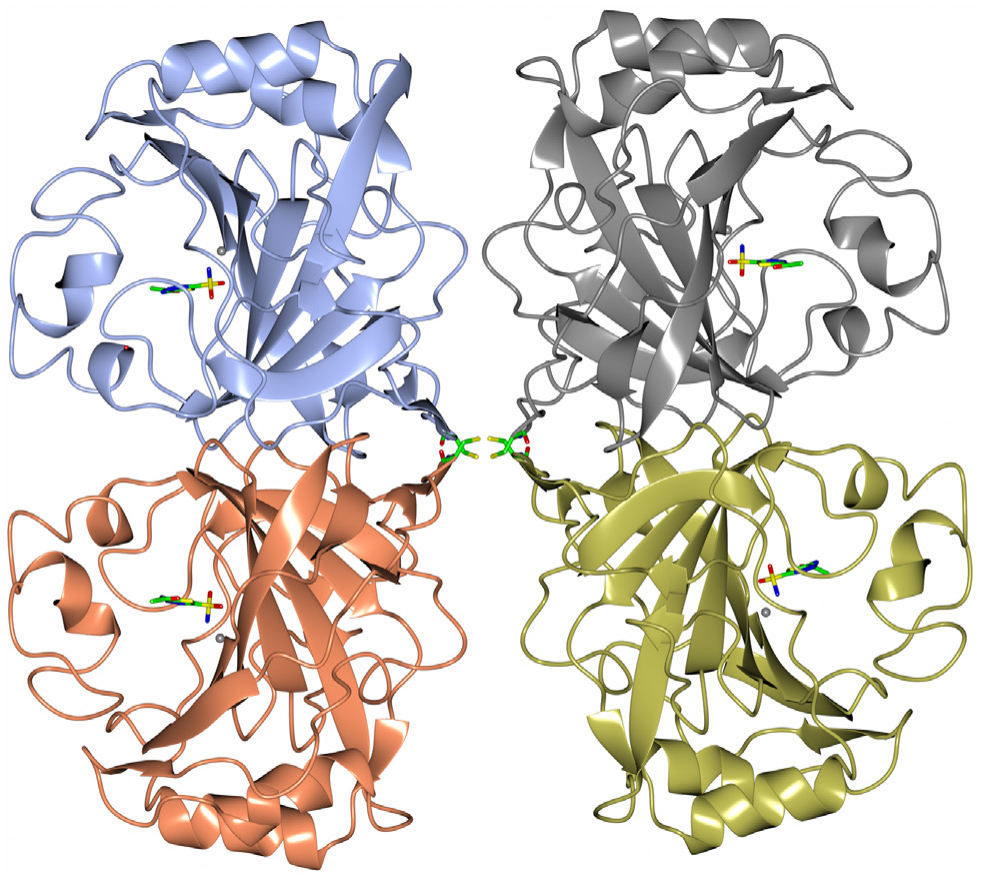
**A diagram of the thermophilic carbonic anhydrase from *Thermovibrio* showing the tetrameric structure held together by disulfide bonds in the center of the molecule (James et al., 2014 PDB code 4UOV)**.

**Figure 4:**
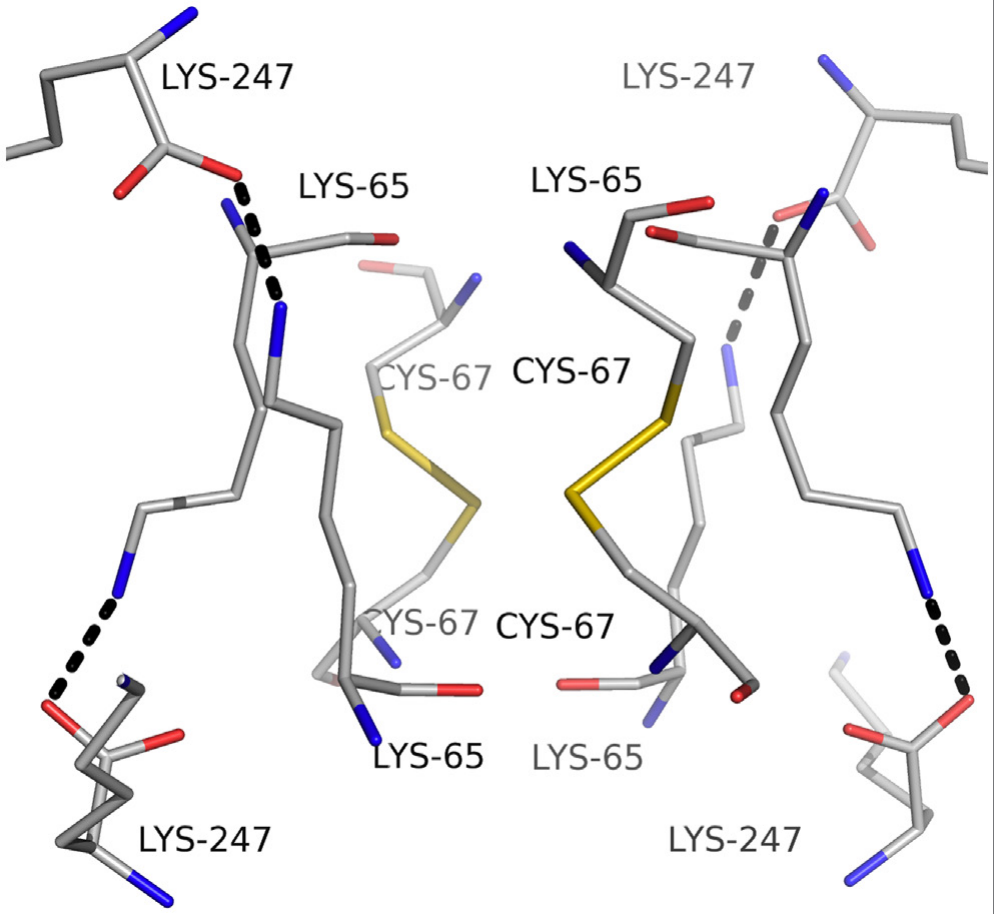
**A diagram showing the unusual structural feature of disulfide bonds in the center of the *Thermovibrio* carbonic anhydrase which are shielded by lysine amino acid residues (James et al., 2014, PDB code 4UOV)**.

Carbonic anhydrase enzymes catalyze the reversible hydration of carbon dioxide to bicarbonate. A stable robust α-carbonic anhydrase has been identified in the thermophilic bacterium *Thermovibrio ammonificans*. The enzyme has been cloned and overexpressed in *E. coli*. This protein has been characterized both biochemically and structurally (James et al., 2014). The crystal structure of this enzyme has been determined in its native form and in two complexes with bound inhibitors. It is unusual since it forms a tetrameric structure rather than the dimer reported for some previously studied related enzymes. The *Thermovibrio* enzyme is stabilized by a unique core in the center of the molecule formed by two intersubunit disulfide bonds and a single lysine residue from each monomer (Figures 3 and 4). The structure of this central core region protects the intersubunit disulfide bonds from reduction. The enzyme is located in the endoplasmic reticulum of *Thermovibrio* as evidenced by the presence of an N-terminal signal peptide. When the recombinant protein is oxidized to mimic the natural environment of the periplasmic space, it shows an increase in thermostability and retains 90% of its activity after incubation at 70°C for 1 h. These properties make it a good candidate for commercial carbon dioxide capture. Another thermophilic bacterial α-carbonic anhydrase has been described from *Sulfurihydrogenibium yellowstonense*. This carbonic anhydrase is also thermostable and is a dimer stabilized by ionic networks (Di Fiore et al., 2013).

The thermophilic bacteria appear to contain the high-activity α-carbonic anhydrase enzymes that have a similar structure to the well-studied bovine carbonic anhydrase enzyme, whereas the archaea have carbonic anhydrases that have different structures and mechanisms. There are six distinct families of CAs (α, β, γ, δ, ζ, and η) (Smith et al., 1999; Guler et al., 2015). The amino acid sequences are conserved between each family; however, there is no sequence or structural similarity between the different families. The carbonic anhydrase activity requires the presence of a catalytic zinc ion which is coordinated to either histidine or cysteine amino acids depending on the class of the enzyme (Silverman and Lindskog, 1988).

### A Thermophilic Transaminase Enzyme from *Sulfolobus solfataricus*

The transaminase enzymes are important biocatalysts for the pharmaceutical industries since they produce chiral amines which are components of a range of different drug molecules. The archaeon *S. solfataricus* has been found to be an interesting source of a thermostable transaminase enzyme of the group IV Pfam group (Sayer et al., 2012). This pyridoxal phosphate (PLP)-containing enzyme is involved in the non-phosphorylated pathway for serine synthesis which is not found in bacteria but is found in animals and plants. The *Sulfolobus* transaminase carries out the conversion of l-serine and pyruvate to 3-hydroxypyruvate and alanine. It also has activity towards methionine, asparagine, glutamine, phenylalanine, histidine, and tryptophan and can be used in a cascade reaction with a C–C bond making enzyme, transketolase, for the synthesis of optically pure drug intermediates (Chen et al., 2006).

**Figure 5:**
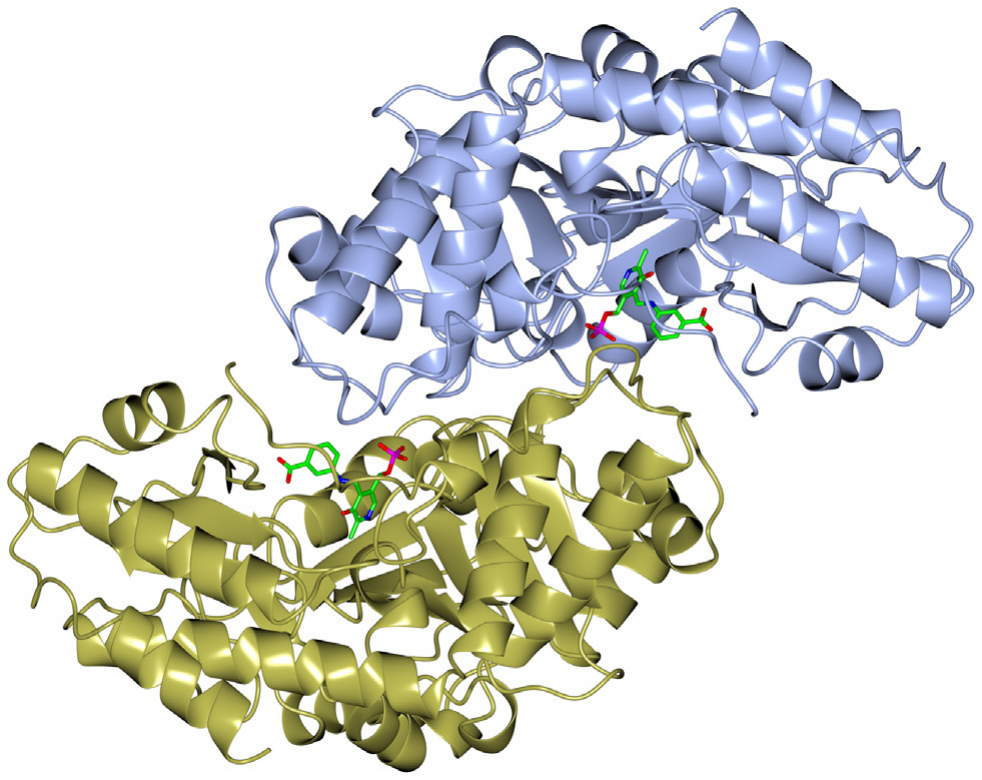
**A diagram of the structure of the *Sulfolobus* transaminase dimer showing the cofactor pyridoxal phosphate (PLP) forming an irreversible complex with the inhibitor gabaculine, shown in stick mode in the two active sites (Sayer et al., 2012, PDB code 3ZRP)**.

The dimeric thermophilic archaeal transaminase enzyme structure has been solved in the holo form of the enzyme and in complex with an inhibitor gabaculine and in a substrate complex with phenolpyruvate, the keto product of phenylalanine (Sayer et al., 2012). Figure 5 shows a cartoon diagram of the dimeric *S. solfataricus* transaminase with an inhibitor bound to the cofactor PLP in the two active sites. The structural studies of this enzyme have given some insight into the conformational changes around the active site occurring during catalysis and have helped to understand the enzyme’s substrate specificity (Sayer et al., 2012). How different members of the PLP enzyme family are able to accept a variety of substrates is vitally important to understand for the use of these enzymes in commercial applications.

The *Sulfolobus* transaminase is relatively thermostable for 10 min at 70°C and at pH 6.5. Features of the archaeal enzyme that relate to its increased stability when compared with a mesophilic related yeast enzyme show that the *Sulfolobus* enzyme has 21 salt bridges compared to 10 in the mesophilic enzyme including several three to four amino acid networks which offer increased stability. There is a C-terminal extension in the *Sulfolobus* enzyme and shorter surface loops which are all general features that are found in thermophilic enzymes. The *Sulfolobus* transaminase dimer interface is unusual being hydrophobic in nature with few ionic interactions which are generally associated with more thermophilic archaeal enzymes. This *Sulfolobus* serine transaminase is the first example of a thermophilic archaeal serine transaminase to be studied structurally and is shown to have properties that meet the requirements for the commercial application of the enzyme in biocatalysis.

### New Epoxide Hydrolases from Extremophilic Metagenomes

An important enzyme activity of interest to the pharmaceutical industry is the ability to catalyze the hydrolysis of an oxirane (epoxide) ring by addition of a molecule of water to form a vicinal diol as a product (Widersten et al., 2010; Kotik et al., 2012). The enzymes that can carry out this reaction are ubiquitously expressed in all living organisms and they play an important physiological role in the detoxification of reactive xenobiotics or endogenous metabolites and in the formation of biologically active mediators. The epoxide hydrolases are already used for the production of optically pure epoxides and diols which are important synthons for the preparation of fine chemicals and drugs, for example, the chiral precursors of β-blockers (Kong et al., 2014; Nestl et al., 2014). The epoxide hydrolase enzymes fall into two classes with completely different 3D structures, the α/β hydrolase fold class and the limonene class (LEH) of which only few have been fully characterized. The LEH enzyme active site contains three residues (Asp, Arg, and Asp) that have been proposed to act in a concerted fashion to activate a water molecule which is able to open the epoxide ring without the formation of a covalently bound alkyl-enzyme intermediate (Arand et al., 2003; Hopmann et al., 2005).

**Figure 6:**
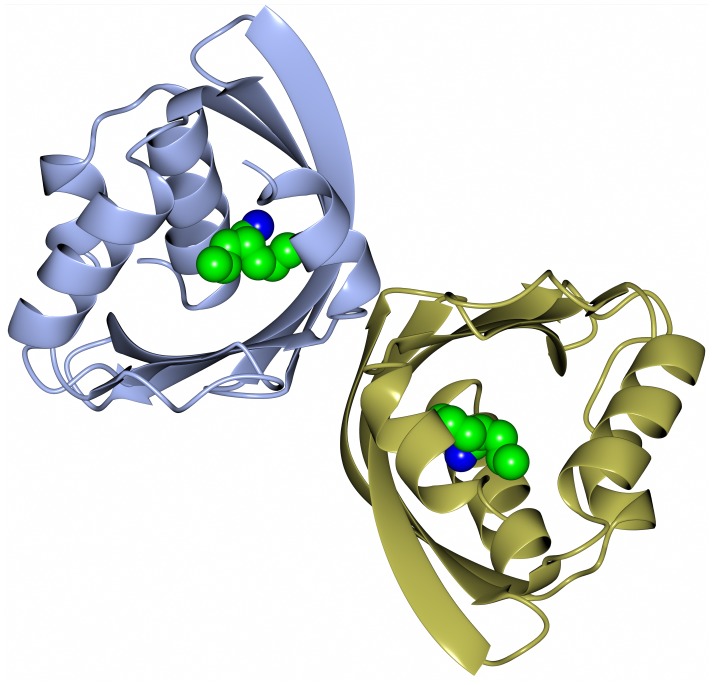
**A diagram showing the thermophilic limonene epoxide hydrolase isolated from the metagenomic sample collected from hot springs in Russia**. The inhibitor, valpromide, is bound into the active site and is shown in sphere mode (Ferrandi et al., 2015a, PDB code 5AIH).

Recently, as part of a thermophilic metagenomic project, two new thermostable epoxide hydrolases of the limonene class have been discovered. The metagenomes were isolated in Russia and China from hot terrestrial environments growing at 46°C and 55°C and at neutral pH. A bioinformatic approach was used to identify the genes coding for these industrially important enzymes which have been cloned and overexpressed in *E. coli*. The resultant proteins have been fully characterized as far as their biochemical properties, specificity, stereoselectivity, and crystal structure (Ferrandi et al., 2015a). The structure of the LEH from the Russian metagenome is shown in Figure 6. The new LEH enzymes have also been further evaluated and used in pilot-scale biotransformations for industrial applications (Ferrandi et al., 2015b).

## Dehalogenase Enzymes from Extremophilic Bacteria and Archaea

### l-Haloacid Dehalogenase from the Thermophilic Archaeon *Sulfolobus tokadii*

A thermophilic dehalogenase enzyme of industrial interest is found in the archaeon *Sulfolobus tokadaii*. This l-haloacid dehalogenase enzyme has been cloned and overexpressed in *E. coli* and has been characterized biochemically and structurally (Rye et al., 2007, 2009). This enzyme has applications for chiral halo-carboxylic acid production and bioremediation. Chiral halo-carboxylic acids are important intermediates in the fine chemical/pharmaceutical industries. The *Sulfolobus* dehalogenase enzyme has the potential to resolve racemic mixtures of bromocarboxylic acids and is able to catalyze the conversion of 2-halo-carboxylic acids to the corresponding hydroxyalkanoic acids. It has been shown to display activity towards longer chain substrates than the bacterial *Xanthomonas autotrophicus* dehalogenase (Van der Ploeg et al., 1991) with activity seen towards 2-chlorobutyric acid due to a more accessible active site (Rye et al., 2009). The enzyme has a maximum activity at 60°C and a half-life of over an hour at 70°C. It is stabilized by a salt bridge and hydrophobic interactions on the subunit interface, helix capping, a more compact subdomain than related enzymes, and shortening of surface loops. A related hyperthermophilic *Pyrococcus* dehalogenase (29% sequence identity) whose structure is available from a structural genomics project is a monomeric enzyme stabilized by a disulfide bond (Arai et al., 2006).

### Dehalogenases from the Marine Environment

The marine environment has been recognized as a potential source of novel enzymes (Trincone, 2011). A novel l-haloalkane dehalogenase has been biochemically and structurally characterized from the psychrophilic bacterium *Psychromonas ingrahamii* (Novak et al., 2013a). This organism was originally isolated in 1991 from Elson Lagoon, Point Barrow Alaska. Original samples were collected from the sea ice interface, where temperatures can reach −10°C (Breezee et al., 2004). The *P. ingrahamii* is a non-motile, large, rod-shaped bacterium that can utilize glycerol as a sole carbon source. The *P. ingrahamii* genome has been shown to encode numerous genes that synthesize polysaccharides and betaine choline. These compounds are thought to aid survival of the organism at low temperatures (Riley et al., 2008). Studies into the structural properties of proteins from psychrophilic organisms have shown they have enhanced structural flexibility at low temperatures (Feller, 2003; Feller and Gerday, 2003). The fine balance between activity, stability, and flexibility of proteins which control enzyme kinetics has been reviewed by Georlette et al. (2004). When proteins from psychrophiles are compared with the equivalent proteins from mesophiles, they show a decrease in the number of ionic interactions, decreased number of hydrogen bonds, and fewer hydrophobic amino acid residues. The l-haloacid dehalogenase from *P. ingrahamii* has been cloned and overexpressed in *E. coli*. The recombinant protein has been biochemically and structurally characterized and compared with mesophilic and thermophilic l-haloacid dehalogenases. It shows activity towards monobromoacetic (100%), monochloroacetic acid (62%), *S*-chloropropionic acid (42%), *S*-bromopropionic acid (31%), dichloroacetic acid (28%), and 2-chlorobutyric acid (10%). The l-haloacid dehalogenase has highest activity towards substrates with shorter carbon chain lengths (≤C3), without preference towards a chlorine or bromine at the α-carbon position. The enzyme has an optimal temperature for activity at 45°C and retains 70% of its activity after being incubated at 65°C for 90 min. The enzyme is relatively stable in organic solvents and therefore shows mesophilic properties despite being isolated from a psychrophilic bacterium. The relatively high thermal stability and optimal temperature for activity is surprising for an enzyme isolated from a psychrophilic bacterium. The thermal stability results show that the enzyme is stable beyond its catalytic temperature optimum. The “equilibrium model” has previously been described to explain the difference between apparent temperature optimum and higher thermostability for a range of psychrophilic, mesophilic, and thermophilic enzymes (Lee et al., 2007). This model includes an inactivated state of the enzyme at temperatures above the optimally active form, which are in reversible equilibrium. At sufficiently higher temperatures, the folded but inactive form of the enzyme can undergo irreversible thermal inactivation to the denatured state (Daniel and Danson, 2010).

Many reported psychrophilic enzymes have highest catalytic efficiency at low temperatures and have low thermal stability. However, some psychrophilic enzymes have been shown to denature at higher temperatures than that they appear to be inactive. For example, the *Pseudoalteromonas haloplanktis* DNA ligase is optimally active below 20°C, inactive above 25°C, but is not fully denatured until 35°C (Georlette et al., 2003). Also the citrate synthase from an Antarctic bacterium has been shown to decrease in enzyme activity at temperatures above its temperature optimum; however, this is not due to thermal denaturation of the enzyme since the activity loss can be reversed as the temperature decreases (Gerike et al., 1997, 2001). Further studies on psychrophilic enzymes will help to understand how these enzymes are adapted to function at low temperatures.

An homology model of the marine l-haloacid dehalogenase has been built based on the crystal structure of related enzymes. The active site pocket of the *P. ingrahamii* model and the *S. tokodaii* and *Burkholderia cepacia* enzymes are highly similar, with almost all residues in similar conformations. The observed thermostability of the enzyme is consistent with the conclusions drawn from homology modeling where no obvious psychrophilic adaptations were observed. At the *in vitro* optimal growth temperature of *P. ingrahamii*, l-haloacid dehalogenase would not be active. This could indicate that the enzyme has been acquired by horizontal gene transfer. This solvent-resistant and stable l-haloacid dehalogenase from *P. ingrahamii* has potential to be used as a biocatalyst in industrial processes.

**Figure 7:**
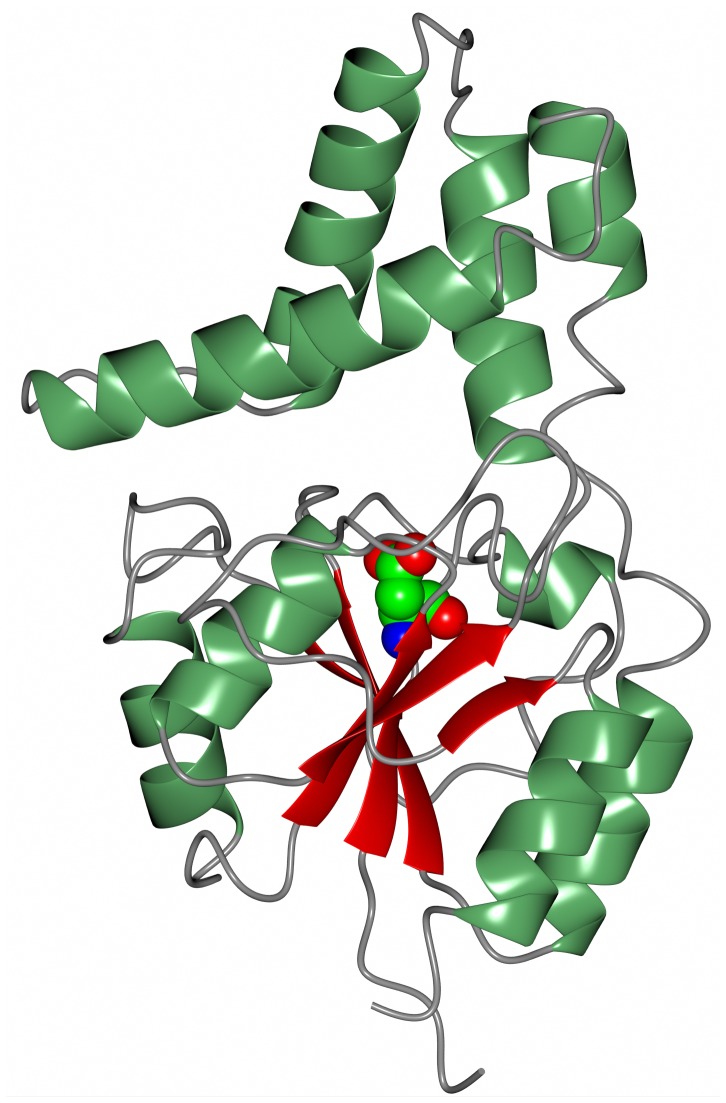
**A diagram of the Rhodobacteraceae l-haloacid dehalogenase showing the smaller cap domain at the top of the molecule and the Rossmann-like fold domain at the bottom**. The catalytic aspartic acid is shown in sphere mode at the interface between the two domains (Novak et al., 2013b, PDB code 2YML).

Another novel marine dehalogenase from the Rhodobacteraceae family has been isolated from a polychaeta worm collected from Tralee beach, Argyll, UK. The enzyme tested positive for l-haloacid dehalogenase activity towards l-monochloropropionic acid (Novak et al., 2013b). A diagram of the overall two-domain structure is shown in Figure 7 with the active site aspartic acid highlighted in the cleft between the domains. The active site of this dehalogenase shows significant differences from previously studied l-haloacid dehalogenases. The asparagine and arginine residues shown to be essential for catalytic activity in other l-haloacid dehalogenases are not present in this enzyme. The histidine residue that replaces the asparagine residue as shown in the structure was coordinated by a conformationally strained glutamate residue that replaces a conserved glycine residue. The His/Glu dyad is positioned for deprotonation of the catalytic water which attacks the ester bond in the reaction intermediate. The catalytic water in this novel enzyme is shifted by from its position in other l-haloacid dehalogenases. A similar His/Glu or Asp dyad is known to activate the catalytic water in haloalkane dehalogenases. The novel enzyme represents a new member within the l-haloacid dehalogenase family and appears to have evolved with properties of a mixture of a haloalkane dehalogenase and a haloacid dehalogenase, and it has the potential to be used as a commercial biocatalyst. It is not unusual to find such novel enzymes in extremophilic microorganisms.

The use of psychrophilic enzymes in industrial processes allows instability issues with reactants and products to be avoided and results in a reduction in cost because of the lower energy consumption. High catalytic efficiency at low temperatures makes psychrophilic enzymes attractive for use in biocatalytic processes (Gomes and Steiner, 2004; Novak and Littlechild, 2013).

## Conclusion

Extremophilic enzymes are becoming an important source of new industrially robust biocatalysts. The use of nature’s biodiversity provides an ever increasing resource of new genomes and metagenomes to identify useful activities which can carry out a variety of chemical biotransformations of commercial interest. The activity of the enzymes can be identified by both bioinformatic techniques and screening of expression libraries. The enzymes can be cloned and overexpressed in easily grown hosts such as *E. coli* allowing access to sufficient quantities of the purified enzymes for detailed biochemical and structural characterization. The scale-up of the enzyme production required for commercial applications can be carried out by using a fungal host system that allows export of the proteins into the growth medium for easy downstream processing, if appropriate. The cost of the enzyme biocatalyst must be matched to the value of the end product. Higher value optically pure pharmaceutical intermediates which are used as building blocks for drug intermediates will allow a higher enzyme price than enzymes used for the production of bulk chemicals, additives for domestic products, food production, or biomass degradation processes. The stability of the biocatalyst is also an economic issue since if the enzyme is sufficiently robust under the industrial conditions it can be used for repeated cycles of the biocatalytic process thereby saving money. The use of enzymes isolated from extremophilic microorganisms offers the opportunity to access enzymes that are stable in a variety of different conditions such as high temperatures, low temperatures, high salt concentrations, high pressure, extremes of pH, and often a combination of these properties, which can make them more suited to the industrial environments.

The use of enzymes in “white biotechnology” is expected to grow with biobased materials and chemicals from emerging technologies predicted to rise globally to over 7.4 million metric tons in 2018 (Lux Research Analysis, www.luxresearchinc.com). Each industrial process is different and the correct biocatalyst needs to be identified and optimized for the industrial application. Many enzyme families have not realized their potential in this area and remain to be discovered. Enzymes that can catalyze reactions with non-natural substrates and under non-physiological conditions, which are often used in industry, can be found in the extremophile environment. Although the techniques available for enzyme engineering have improved recently, the enzyme discovery and optimization process is still a limiting factor for the adoption of new biobased industrial processes.

## Conflict of Interest Statement

The authors declare that the research was conducted in the absence of any commercial or financial relationships that could be construed as a potential conflict of interest.

## References

[B1] Andreeva A. Howorth D. Chothia C. Kulesha E. Murzin A. G. (2014). SCOP2 prototype: a new approach to protein structure mining. Nucleic Acids Res. 42, D310–D314. 10.1093/nar/gkt1242 24293656 PMC3964979

[B2] Arai R. Kukimoto-Niino M. Kuroishi C. Bessho Y. Shirouzu M. Yokoyama S. (2006). Crystal structure of the probable haloacid dehalogenase PH0459 from *Pyrococcus horikoshii* OT3. Protein Sci. 15, 373–377. 10.1110/ps.051922406 16385007 PMC2242451

[B3] Arand M. Hallberg B. M. Zou J. Bergfors T. Oesch F. van der Werf M. J. (2003). Structure of *Rhodococcus erythropolis* limonene-1,2-epoxide hydrolase reveals a novel active site. EMBO J. 22, 2583–2592. 10.1093/emboj/cdg275 12773375 PMC156771

[B4] Bains J. Kaufman L. Farnell B. Boulanger M. J. (2011). A product analog bound form of 3-oxoadipate-enol-lactonase (PcaD) reveals a multifunctional role for the divergent cap domain. J. Mol. Biol. 406, 649–658. 10.1016/j.jmb.2011.01.007 21237173

[B5] Baxter S. Royer S. Grogan G. Brown F. Holt-Tiffin K. Taylor I. (2012). An improved racemase/acylase biotransformation for the amino acids. J. Am. Chem. Soc. 134, 19310–19313. 10.1021/ja305438y 23130969

[B6] Bommarius A. S. Schwarm M. Drauz K. (1998). Biocatalysis to aminoacid-based chiral pharmaceuticals: examples and perspectives. J. Mol. Catal. B Enzym. 5, 1–11. 10.1016/S1381-1177(98)00009-5

[B7] Breezee J. Cady N. Staley J. T. (2004). Subfreezing growth of the sea ice bacterium *Psychromonas ingrahamii*. Microb. Ecol. 47, 300–304. 10.1007/s00248-003-1040-9 14994177

[B8] Cheeseman J. D. Tocilj A. Park S. Schrag J. D. Kazlauskas R. J. (2004). Structure of an aryl esterase from *Pseudomonas fluorescens*. Acta Crystallogr. D60, 1237–1243. 10.1107/S0907444904010522 15213385

[B9] Chen B. H. Sayar A. Kaulmann U. Dalby P. A. Ward J. M. Woodley J. M. (2006). Reaction modelling and simulation to assess the integrated use of transketolase and ω-transaminase for the synthesis of an aminotriol. Biocatal. Biotransformation 24, 449–457. 10.1080/10242420601068668

[B10] Daniel R. M. Danson M. J. (2010). A new understanding of how temperature affects the catalytic activity of enzymes. Trends Biochem. Sci. 35, 584–591. 10.1016/j.tibs.2010.05.001 20554446

[B11] Di Fiore A. Capasso C. De Luca V. Monti S. M. Carginale V. Supuran C. T. (2013). X-ray structure of the first ‘extremo-α-carbonic anhydrase’, a dimeric enzyme from the thermophilic bacterium *Sulfurihydrogenibium yellowstonense* YO3AOP1. Acta Crystallogr. D69, 1150–1159. 10.1107/S0907444913007208 23695259

[B12] Drauz K. (1997). Chiral amino acids: a versatile tool in the synthesis of pharmaceuticals and fine chemicals. Int J Chem Chimia 51, 310–314.

[B13] Feller G. (2003). Molecular adaptations to cold in psychrophilic enzymes. Cell. Mol. Life Sci. 60, 648–662. 10.1007/s00018-003-2155-3 12785714 PMC11138853

[B14] Feller G. Gerday C. (2003). Psychrophilic enzymes: hot topics in cold adaption. Nat. Rev. Microbiol. 1, 200–208. 10.1038/nrmicro773 15035024

[B15] Ferrandi E. E. Sayer C. Isupov M. N. Annovazzi C. Marchesi C. Iacobone G. (2015a). Discovery and characterization of thermophilic limonene-1,2-epoxide hydrolases from hot spring metagenomics libraries. FEBS J. 282, 2879–2894. 10.1111/febs.13328 26032250

[B16] Ferrandi E. Marchesi C. Annovazzi C. Riva S. Monti D. Wohlgemuth R. (2015b). Efficient epoxide hydrolase-catalyzed resolutions of (+)- and (-)-*cis*/*trans*-limonene oxides. ChemCatChem10.1002/cctc.201500608

[B17] Georlette D. Blaise V. Collins T. D’Amico S. Gratia E. Hoyoux A. (2004). Some like it cold: biocatalysis at low temperatures. FEMS Microbiol. Rev. 28, 25–42. 10.1016/j.femsre.2003.07.003 14975528

[B18] Georlette D. Damiens B. Blaise V. Depiereux E. Uversky V. N. Gerday C. (2003). Structural and functional adaptations to extreme temperatures in psychrophilic, mesophilic and thermophilic DNA ligases. J. Biol. Chem. 278, 37015–37023. 10.1074/jbc.M305142200 12857762

[B19] Gerike U. Danson M. J. Hough D. W. (2001). Cold-active citrate synthase: mutagenesis of active-site residues. Protein Eng. 14, 655–661. 10.1093/protein/14.9.655 11707611

[B20] Gerike U. Danson M. J. Russell N. J. Hough D. W. (1997). Sequencing and expression of the gene encoding a cold active citrate synthase from an Antarctic bacterium, strain DS2 3R. Eur. J. Biochem. 248, 49–57. 10.1111/j.1432-1033.1997.00049.x 9310359

[B21] Gomes J. Steiner W. (2004). The biocatalytic potential of Extremophiles and Extremozymes. Food Technol. Biotechnol. 42, 223–235.

[B22] Gonsalvez I. S. Isupov M. Littlechild J. A. (2001). Crystallization and preliminary X-ray analysis of a gamma-lactamase. Acta Crystallogr. D57, 284–286. 10.1107/S0907444900016838 11173481

[B23] Guler O. O. Capasso C. Supuran C. T. (2015). A magnificent enzyme superfamily: carbonic anhydrases, their purification and characterization. J. Enzyme Inhib. Med. Chem. 10.3109/14756366.2015.1059333 26118417

[B24] Hickey A. M. Ngamsom B. Wiles C. Greenway G. M. Watts P. Littlechild J. A. (2009). A microreactor for the study of biotransformations by a cross-linked gamma-lactamase enzyme. Biotechnol. J. 4, 510–516. 10.1002/biot.200800302 19291707

[B25] Holt K. (2004). Biocatalysis and chemocatalysis – a powerful combination for the preparation of enantiomerically pure α-amino acids. Pharmachem 3, 2–4. 10.1002/chin200542274

[B26] Hopmann K. H. Hallberg B. M. Himo F. (2005). Catalytic mechanism of limonene epoxide hydrolase, a theoretical study. J. Am. Chem. Soc. 127, 14339–14347. 10.1021/ja050940p 16218628

[B27] James P. Isupov M. N. Sayer C. Saneei V. Berg S. Lioliou M. (2014). The structure of a tetrameric α-carbonic anhydrase from *Thermovibrio ammonificans* reveals a core formed around intermolecular disulfides that contribute to its thermostability. Acta Crystallogr. D70, 2607–2618. 10.1107/S1399004714016526 25286845

[B28] Kobayashi M. Fujiwara Y. Goda M. Komeda H. Shimizu S. (1997). Identification of active sites in amidase: evolutionary relationship between amide bond- and peptide bond-cleaving enzymes. Proc. Natl. Acad. Sci. U.S.A. 94, 11986–11991. 10.1073/pnas.94.22.11986 9342349 PMC23678

[B29] Kong X.-D. Ma Q. Zhou J. Zeng B.-B. Xu J.-H. (2014). A smart library of epoxide hydrolase variants and the top hits for synthesis of (*S*)-β-blocker precursors. Angew. Chem. Int. Ed. 53, 6641–6644. 10.1002/anie.201402653 24841567

[B30] Kotik M. Archelas A. Wohlgemuth R. (2012). Epoxide hydrolases and their application in organic synthesis. Curr. Org. Chem. 16, 451–482. 10.2174/138527212799499840

[B31] Lee C. K. Daniel R. M. Shepherd C. Saul D. Cary S. C. Danson M. J. (2007). Eurythermalism and temperature dependence of enzyme activity. FASEB J. 8, 1934–1941. 10.1096/fj.06-7265com 17341686

[B32] Line K. Isupov M. N. Littlechild J. A. (2004). The crystal structure of a (−) γ-lactamase from an *Aureobacterium* species reveals a tetrahedral intermediate in the active site. J. Mol. Biol. 338, 519–532. 10.1016/j.jmb.2004.03.001 15081810

[B33] Littlechild J. James P. Novak H. Sayer C. (2013). “Mechanisms of thermal stability adopted by thermophilic proteins and their use in white biotechnology,” in Thermophilic Microbes in Environmental and Industrial Biotechnology, Biotechnology of Thermophiles, 2nd Edn, eds Satyanarayana T. Littlechild J. Kawarabayasi Y. (London: Springer Publishers), 481–507.

[B34] Nestl B. M. Hammer S. C. Nebel B. A. Hauer B. (2014). New generation of biocatalysts for organic synthesis. Angew. Chem. Int. Ed. 53, 3070–3095. 10.1002/anie.201302195 24520044

[B35] Ngamsom B. Hickey A. M. Greenway G. M. Littlechild J. A. Watts P. Wiles C. (2010). Development of a high throughput screening tool for biotransformations utilising a thermophilic l-aminoacylase enzyme. J. Mol. Catal. B Enzym. 63, 81–86. 10.1016/j.molcatb.2009.12.013

[B36] Novak H. Littlechild J. (2013). “Marine enzymes for biocatalysis. Sources, biocatalytic characteristics and bioprocesses of marine enzymes,” in Marine Enzymes with Applications for Biosynthesis of Fine Chemicals, ed. Trincone A. (Cambridge: Woodhead Publishing Series in Biomedicine), 89–102.

[B37] Novak H. R. Sayer C. Panning J. Littlechild J. A. (2013a). Characterisation of an l-haloacid dehalogenase from the marine psychrophile *Psychromonas ingrahamii* with potential industrial application. Mar. Biotechnol. 6, 695–705. 10.1007/s10126-013-9522-3 23949008

[B38] Novak H. R. Sayer C. Isupov M. N. Paszkiewicz K. Gotz D. Mearns-Spragg A. (2013b). Marine *Rhodobacteraceae* l-haloacid dehalogenase contains a novel His/Glu dyad which could activate the catalytic water. FEBS J. 280, 1664–1680. 10.1111/febs.12177 23384397

[B39] Riley M. Staley J. T. Danchin A. Wang T. Z. Brettin T. S. Hauser L. J. (2008). Genomics of an extreme psychrophile, *Psychromonas ingrahamii*. BMC Genomics 9:210. 10.1186/1471-2164-9-210 18460197 PMC2405808

[B40] Rye C. A. Isupov M. N. Lebedev A. A. Littlechild J. A. (2007). An order-disorder twin crystal of l-2-haloacid dehalogenase from *Sulfolobus tokodaii*. Acta Crystallogr. D63, 926–930. 10.1107/S0907444907026315 17642519

[B41] Rye C. A. Isupov M. N. Lebedev A. A. Littlechild J. A. (2009). Biochemical and structural studies of a l-haloacid dehalogenase from the thermophilic archaeon *Sulfolobus tokodaii*. Extremophiles 13, 179–190. 10.1007/s00792-008-0208-0 19039518

[B42] Sayer C. Bommer M. Ward J. M. Isupov M. N. Littlechild J. A. (2012). Crystal structure and substrate specificity of the thermophilic serine:pyruvate aminotransferase from *Sulfolobus solfataricus*. Acta Crystallogr. D68, 763–772. 10.1107/S0907444912011274 22751661

[B43] Sayer C. Isupov M. N. Bonch-Osmolovskaya E. Littlechild J. A. (2015). Structural studies of a thermophilic esterase from a new *Planctomycetes* species, *Thermogutta terrifontis*. FEBS J. 282, 2846–2857. 10.1111/febs.13326 26011036

[B44] Silverman D. N. Lindskog S. (1988). The catalytic mechanism of carbonic anhydrase: implications of a rate-limiting protolysis of water. Acc. Chem. Res. 21, 30–36. 10.1021/ar00145a005

[B45] Singleton M. Isupov M. Littlechild J. (1999). X-ray structure of pyrrolidone carboxyl peptidase from the hyperthermophilic archaeon *Thermococcus litoralis*. Structure 7, 237–244. 10.1016/S0969-2126(99)80034-3 10368293

[B46] Smith K. S. Jakubzick C. Whittam T. S. Ferry J. G. (1999). Carbonic anhydrase is an ancient enzyme widespread in prokaryotes. Proc. Natl. Acad. Sci. U.S.A. 96, 15184–15189. 10.1073/pnas.96.26.15184 10611359 PMC24794

[B47] Tanimoto K. Higashi N. Nishioka M. Ishikawa K. Taya M. (2008). Characterization of thermostable aminoacylase from hyperthermophilic archaeon *Pyrococcus horikoshii*. FEBS J. 275, 1140–1149. 10.1111/j.1742-4658.2008.06274.x 18248457

[B48] Taylor S. J. C. McCague R. Wisdom R. Lee C. Dickson K. Ruecroft G. (1993). Development of the biocatalytic resolution of 2-azabicyclo [2.2.1] hept-5-en-3-one as an entry to single-enantiomer carbocyclic nucleosides. Tetrahedron 4, 1117–1128. 10.1016/S0957-4166(00)80218-9

[B49] Toogood H. S. Brown R. C. Line K. Keene P. A. Taylor S. J. C. McCague R. (2004). The use of a thermostable signature amidase in the resolution of the bicyclic synthon (rac)-γ-lactam. Tetrahedron 60, 711–716. 10.1016/j.tet.2003.11.064

[B50] Toogood H. S. Hollingsworth E. J. Brown R. C. Taylor I. N. Taylor S. J. McCague R. (2002a). A thermostable l-aminoacylase from *Thermococcus litoralis*: cloning, overexpression, characterization, and applications in biotransformations. Extremophiles 6, 111–122. 10.1007/s007920100230 12013431

[B51] Toogood H. S. Taylor I. N. Brown R. C. Taylor S. J. C. McCague R. Littlechild J. A. (2002b). Immobilisation of the thermostable l-aminoacylase from *Thermococcus litoralis* to generate a reusable industrial biocatalyst. Biocatal. Biotransformation 20, 241–249. 10.1080/10242420290029472

[B52] Trincone A. (2011). Marine biocatalysts: enzymatic features and applications. Mar. Drugs 9, 478–499. 10.3390/md9040478 21731544 PMC3124967

[B53] Van der Ploeg J. Van Hall G. Janssen D. B. (1991). Characterization of the haloacid dehalogenase from *Xanthobacter autotrophicus* GJ10 and sequencing of the *dhlB* gene. J. Bacteriol. 173, 7925–7933. 1744048 10.1128/jb.173.24.7925-7933.1991 PMC212586

[B54] Widersten M. Gurell A. Lindberg D. (2010). Structure-function relationships of epoxide hydrolases and their potential use in biocatalysis. Biochim. Biophys. Acta 1800, 316–326. 10.1016/j.bbagen.2009.11.014 19948209

[B55] Yin D. L. Bernhardt P. Morley K. L. Jiang Y. Cheeseman J. D. Purpero V. (2010). Switching catalysis from hydrolysis to perhydrolysis in *P. fluorescens* esterase. Biochemistry 49, 1931–1942. 10.1021/bi9021268 20112920 PMC2855817

